# Crosstalk between Spinal Astrocytes and Neurons in Nerve Injury-Induced Neuropathic Pain

**DOI:** 10.1371/journal.pone.0006973

**Published:** 2009-09-11

**Authors:** Wei Wang, Wen Wang, Xiaopeng Mei, Jing Huang, Yanyan Wei, Yayun Wang, Shengxi Wu, Yunqing Li

**Affiliations:** Department of Anatomy, Histology and embryology; K. K. Leung Brain Research Centre, The Fourth Military Medical University, Xi'an, People's Republic of China; Emory University, United States of America

## Abstract

Emerging research implicates the participation of spinal dorsal horn (SDH) neurons and astrocytes in nerve injury-induced neuropathic pain. However, the crosstalk between spinal astrocytes and neurons in neuropathic pain is not clear. Using a lumbar 5 (L5) spinal nerve ligation (SNL) pain model, we testified our hypothesis that SDH neurons and astrocytes reciprocally regulate each other to maintain the persistent neuropathic pain states. Glial fibrillary acidic protein (GFAP) was used as the astrocytic specific marker and Fos, protein of the protooncogene c-*fos*, was used as a marker for activated neurons. SNL induced a significant mechanical allodynia as well as activated SDH neurons indicated by the Fos expression at the early phase and activated astrocytes with the increased expression of GFAP during the late phase of pain, respectively. Intrathecal administration of c-*fos* antisense oligodeoxynucleotides (ASO) or astroglial toxin L-α-aminoadipate (L-AA) reversed the mechanical allodynia, respectively. Immunofluorescent histochemistry revealed that intrathecal administration of c-*fos* ASO significantly suppressed activation of not only neurons but also astrocytes induced by SNL. Meanwhile, L-AA shortened the duration of neuronal activation by SNL. Our data offers evidence that neuronal and astrocytic activations are closely related with the maintenance of neuropathic pain through a reciprocal “crosstalk”. The current study suggests that neuronal and non-neuronal elements should be taken integrally into consideration for nociceptive transmission, and that the intervention of such interaction may offer some novel pain therapeutic strategies.

## Introduction

Peripheral nerve injury often results in neuropathic pain [1] which is essentially intractable to currently available anti-nociceptive therapies. This is in part due to poor understanding on how neuropathic pain is induced and maintained [2]. According to classic pain research, the pain pathway has been described simply as a serial chain of neuronal elements, but immerging research has implicated spinal cord glial cells as key players in the induction and maintenance of neuropathic pain [3]. Due to the immune cell nature of microglia and their ability of releasing the “proinflammatory” cytokines such as interlukin-1 (IL-1), tumor necrosis factor (TNF) and adenosine triphosphate (ATP) when activated [4], [5], [6], [7], [8], microglia receive intensive focus in pain research, especially persistent pain after inflammation or nerve injury [9], [10], [11], [12]. Thanks to decades of pain researchers' endeavors, the underlying mechanisms for microglia-neuron interaction and its contribution to neuropathic pain have almost been clarified [13]. However, the spinal astrocytes, which are more dominant than microglia, are majorly investigated for its supportive and tropic functions for neurons [14], [15] instead of their contributions to neuropathic pain. A few studies suggested the activation of spinal astrocytes during neuropathic pain [3], [13], [16], [17], but it is not clear whether there exists astrocyte-neuron crosstalk just as in the case of microglia, and how this happens [18].

C*-fos*, as a protooncogene, has been shown to act as an inducible transcriptional factor and suggested to regulate the expression of the downstream response genes [6], [19], [20]. Fos protein is rapidly expressed in the spinal dorsal horn neurons following noxious peripheral nerve stimulation [21] and correlates well with nociceptive behaviors [19]. Thus, Fos expression is used to identify nociceptive input activated or excited population of neurons [22], [23], [24].

Astrocytes can sensitively and rapidly respond to various physiological or noxious stimuli with the increased expression of glial fibrillary acidic protein (GFAP), thus be used as a marker for astrocytic activation [25]. Our previous research indicates that GFAP upregulation correlates well with nerve injury-induced neuropathic pain [26]. However, astrocytes cannot generate action potential and are not able to contribute to the nociceptive transmission alone and directly [14]. Hence, a hypothesis is that the activated astrocytes and neurons are “crosstalking” so as to contribute to the neuropathic pain states [27]. However, there still lacks direct evidence for this hypothesis.

To achieve this goal, it is necessary to shut-down the activated astrocytes or neurons to elucidate their individual contribution to neuropathic pain. In the current study, L-α-aminoadipate (L-AA) was used to inactivate astrocytes based on the fact that its disruption of the astrocytic network in amygdala is confined to astrocytes [28], [29]. C-*fos* antisense oligodeoxynucleotides (ASO) was used in our previous studies and the corresponding nociceptive behavioral changes were observed as a consequence of knocking down its protein product [24], [30], [31], [32], thus, it was employed in this study to shut down activated neurons.

In the current study, we observed the temporal activation of neurons and astrocytes along with lumbar 5 spinal nerve ligation (SNL) induced neuropathic pain behaviors by using Fos or GFAP expression, respectively. Then, the effect of intrathecal (*i.t.*) injection of c-*fos* ASO or L-AA on pain behaviors as well as neuronal or astrocytic activation was identified morphologically. The possible “crosstalk” between astrocytes and neurons was investigated by checking the effect of inhibited activation of one type of cells on the other.

## Results

### SNL-induced mechanical allodynia was reversed by c-*fos* ASO or L-AA treatment

SNL-induced neuropathic pain is characterized by ipsilateral mechanical allodynia [33]. Previous studies indicated that SNL produced rapidly appearing (<3 d) and persistent (>3 w) ipsilateral mechanical allodynia shown by decrease of paw withdrawal threshold (PWT) [17], [26].

**Figure 1:**
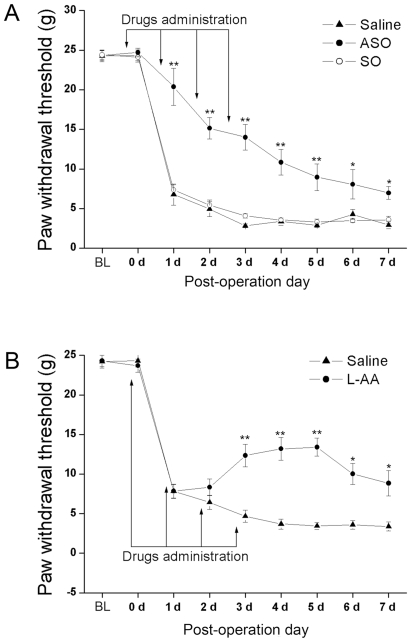
Effects of intrathecal administration of c-*fos* ASO (A) or L-AA (B) on SNL-induced mechanical allodynia. A, SNL rats in saline and c*-fos* SO group demonstrated significant ipsilateral allodynia since 1 d after the surgery. Intrathecal administration of c-*fos* ASO (50 µg/10 µl, starting 4 h prior to the surgery and once a day for a further 3 d following surgery) significantly reversed the ipsilateral allodynia. The preventing effect of ASO weakened gradually but still significant 7 d after SNL B, Intrathecal administration of astroglial toxin L-AA (100 nmol/10 µl, injected daily from 1 d before till 3 d after the surgery) reversed mechanical allodynia only at the late stage. The data are presented as the mean ± S.E.M. * and ** indicate statistically significant difference with *P*<0.05 and *P*<0.01 compared to saline control of each time point, respectively.

C-*fos* ASO treatment significantly reversed mechanical allodynia in 3 d post SNL. The ASO treatment and post operative day (POD) had significant effect on the SNL-induced ipsilateral allodynia [Fig. 1A; two way ANOVA (between subjects factors: Group and Day): Group: F (2,326) = 127.314, *P* = 0.000; Day: F (7,326) = 209.909, *P* = 0.000; Group*Day: F (14,326) = 9.676, *P* = 0.000]. The effect of SNL on the allodynia of saline and SO groups was incubated with time. SNL rats in saline and c*-fos* SO group demonstrated significant ipsilateral allodynia 1 d after the surgery (PWT = 6.8±1.3 g and 7.4±0.6 g on POD 1 for Saline and SO groups, respectively; *Post hoc* test, *P*<0.05, vs corresponding baseline for saline and SO group, respectively) and the ipsilateral allodynia reached a high level on POD 3 and lasted for the whole observing window. There was no significant difference between saline and SO group at every corresponding time point. Treatment with ASO significantly reversed the ipsilateral allodynia on POD 1 (PWT = 6.8±1.3 g, 7.4±0.6 g, 20.4±2.3 g at POD 1 for Saline, SO and ASO group, respectively; *Post hoc* test, *P*<0.01, vs Saline or SO group), and such reversal effect existed for the whole observing window (*Post hoc* test, *P*<0.01, for 2, 3, 4, 5 and *P*<0.05 for 6 and 7 days after SNL, vs Saline or SO group). But the preventing effect weakened gradually. On POD 4, PWT in the Saline group was 3.4±0.4 g, while it was 10.8±1.6 g in the ASO group. And the difference of PWT between groups was much less but still significant 7 d after SNL (Fig. 1A). Thus, SNL itself produced a significant allodynia which was incubated with time; SO treatment did not change the neuropathic pain performance after SNL, while ASO treatment reversed the SNL-induced neuropathic pain with a long-lasting effect.

Treatment with the astrocyte inactivator L-AA, reversed mechanical allodynia only at the late stage. The L-AA treatment and POD had significant effect on the SNL-induced ipsilateral allodynia [Fig. 1B; two way ANOVA (between subjects factors: Group and Day): Group: F (1,216) = 157.194, *P* = 0.000; Day: F (7,216) = 94.389, *P* = 0.000; Group*Day: F (7,216) = 11.567, p = 0.000]. Treatment with L-AA significantly reversed the ipsilateral allodynia on POD 3 (PWT = 4.7±0.8 g and 12.4±1.4 g for Saline and L-AA groups, respectively; *Post hoc* test, *P*<0.01), and such reversal effect existed since POD 3 (*Post hoc* test, *P*<0.01 for POD 4 and 5 and *P*<0.05 for POD 6 and 7, vs saline group). On POD 7, the PWTs for saline and L-AA groups were 3.4±0.5 g and 8.8±1.6 g, respectively. Inactivation of astrocytes by L-AA selectively reversed mechanical allodynia 7 d post nerve injury.

In the sham-operated animals, PWTs was not influenced compared with that of naïve rats. Furthermore, intrathecal injection of c-*fos* ASO, SO or L-AA on sham-operated rats did not change their PWTs (data not shown).

### SNL enhanced Fos and GFAP expression in the ipsilateral spinal dorsal horn

**Figure 2:**
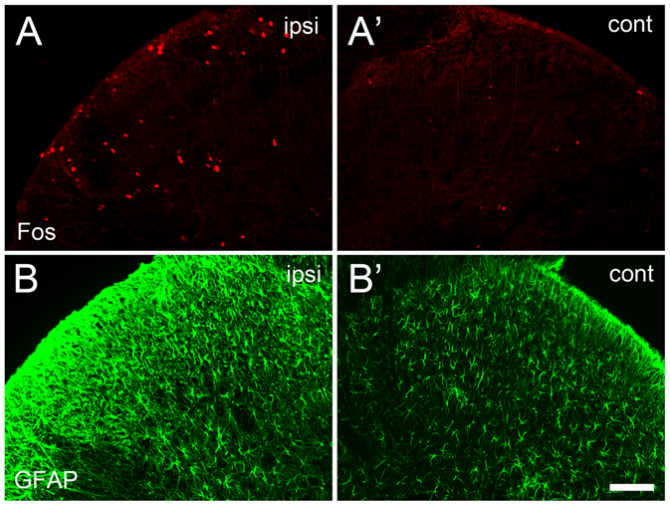
Immunofluorescent staining for Fos and GFAP in the spinal dorsal horn after SNL. A–A', SNL induced a significant up-regulation of Fos expression in the ipsilateral dorsal horn 2 h after SNL. B–B', GFAP immunoreactive astrocytes were significantly activated 1 w after nerve injury in the ipsilateral compared to the contralateral dorsal horn. Scale bar = 200 µm.

**Figure 3:**
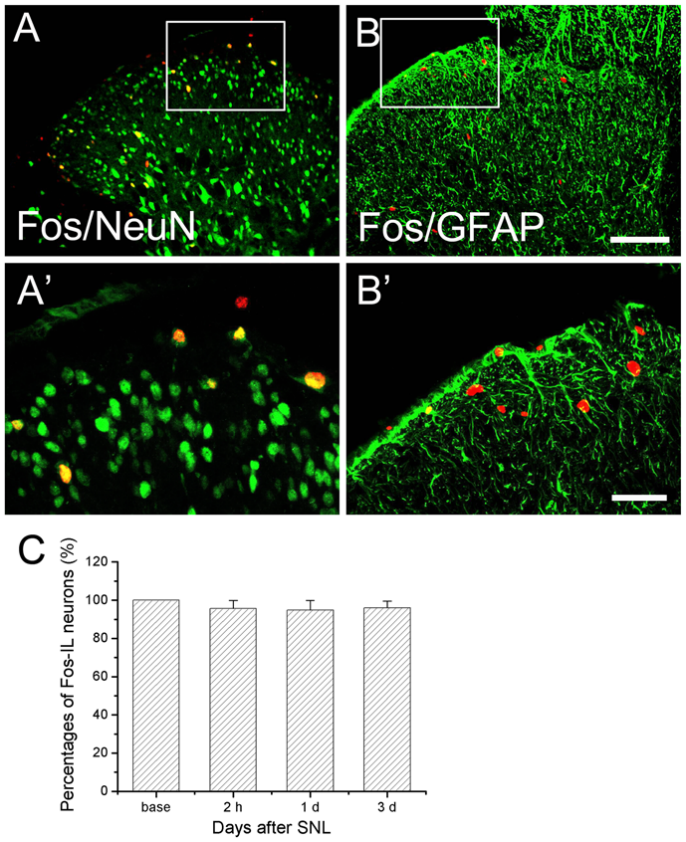
Double immunofluorescence for Fos (red)/NeuN (green) and Fos (red)/GFAP (green) in the ipsilateral dorsal horn 3 d after SNL. A' and B' are the magnified images of the rectangles indicated in the above top panels in A and B, respectively. Double immunofluorescence indicated that few GFAP-IR astrocytes were Fos positive (shown by double arrowheads in B'), while most of them were nuclei of activated neurons (shown by double arrowheads in A'). These activated neurons and astrocytes were mainly distributed in the superficial layer of dorsal horn, and the Fos-positive neurons were surrounded with many GFAP-positive astrocytic processes formed close relationship with each other. The figures shows representative photomicrographs obtained from a minimum of three independent experiments. Fos and NeuN double labeled neurons observed in the ipsilateral superficial dorsal horn were counted and the proportion of double labeling neurons in total Fos positive cells were calculated. The statistical results of these comparisons are presented in C. Scale bar = 200 µm in A, B and 80 µm in A' and B'.

Immunofluorescent histochemistry was employed to demonstrate the Fos expression from different groups (Fig. 2A, A'). To rule out the possible grouping and treatment bias, the number of Fos-immunoreactive (IR) neurons was normalized to that in naïve rats at corresponding time points. SNL induced a significant increase of Fos expression in the ipsilateral but not the contralateral spinal dorsal horn (Fig. 2A, A'). A time-course study showed that the Fos expression reached the highest level 2 h after SNL, then declined gradually, to normal level by 1 w (Fig. 2D, 5D). Almost all of the Fos-IR cells were neurons as indicated by NeuN staining (95% of Fos-IR neurons were also positive for NeuN, Fig. 3). SNL-induced Fos expression was restricted to the neuronal population as the ratio of Fos positive NeuN-IR cells to Fos-IR did not vary too much among different groups nor at different time points. Thus, increased Fos expression was reliably used as an marker for activated neurons but not astrocytes. Meanwhile, most of the Fos-IR neurons were observed in laminae I and II with a few in laminae III and IV and some in laminae V and VI of the dorsal horn (Fig. 2A), suggesting most of them were nociceptive neurons.

**Figure 4:**
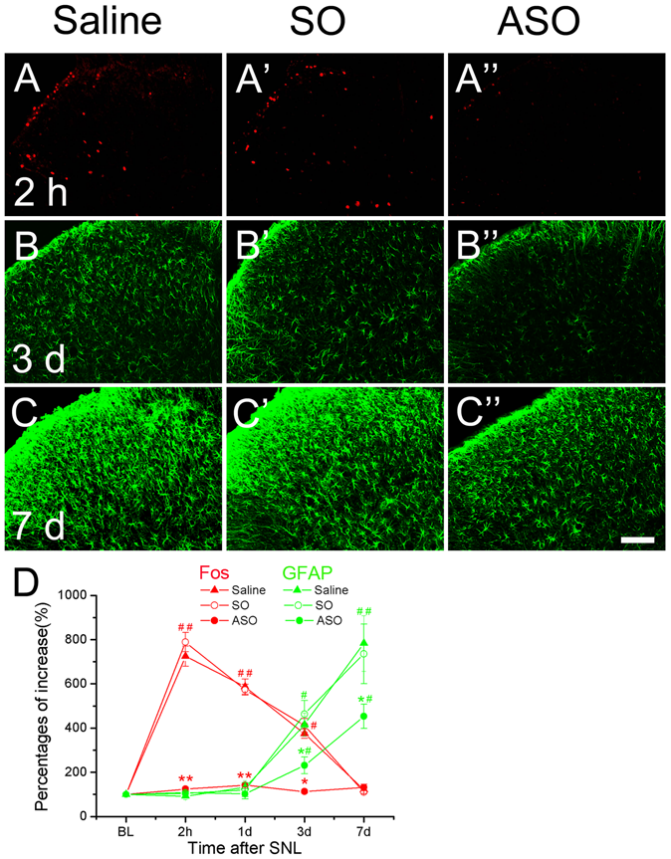
The expressions of Fos and GFAP in the ipsilateral dorsal horn in saline, c-*fos* SO and ASO groups after SNL. Compared to saline or c-*fos* SO treated group, c-*fos* ASO produced a significant inhibitory effect on Fos protein expression (A–A″), as well as on astrocytic activation (B–B″, C–C″) in the spinal dorsal horn. D, Statistics of the time course study. The baseline level of Fos and GFAP was set as 100%, respectively. The changing course of Fos (red curves) or GFAP (green curves) was determined with comparison to the relative baseline value. The relative increase in the number of Fos-positive neurons reached a peak 2 h in the SO and saline group, then fell back quickly to the normal level in 1 w. However, Fos expression was maintained at the baseline level by ASO treatment. The GFAP expression presented a different changing course tendency: increasing since day 3 and keeping a high level 1 w after SNL. Interestingly, the increase of GFAP expression was interfered with by ASO treatment on day 3 and 7. #, *P*<0.05 and # #, *P*<0.01, as compared to the baseline value; *, *P*<0.05 and **, *P*<0.01, as compared to the saline control in the same time point. Scale bar = 200 µm.

Similar to our previous observation [26], SNL induced a marked expression of GFAP in the ipsilateral but not the contralateral dorsal horn (Fig. 2B). A time course study showed that GFAP up-regulation was not evident on POD 1, but became significant on POD 3, and reached a peak on POD 7 after SNL (Fig. 4D, 5D). Double immunofluorescent labeling indicated that few GFAP-IR cells were positive for Fos, while most of them were overlapped nuclei of activated neurons (Fig. 4). These activated astrocytes were mainly distributed in the superficial laminae of dorsal horn ipsilateral to the injury, suggesting a role in the nociceptive transmission. The Fos-IR neurons were surrounded with many GFAP-IR astrocytic processes, forming many close contacts (Fig. 3B').

**Figure 5:**
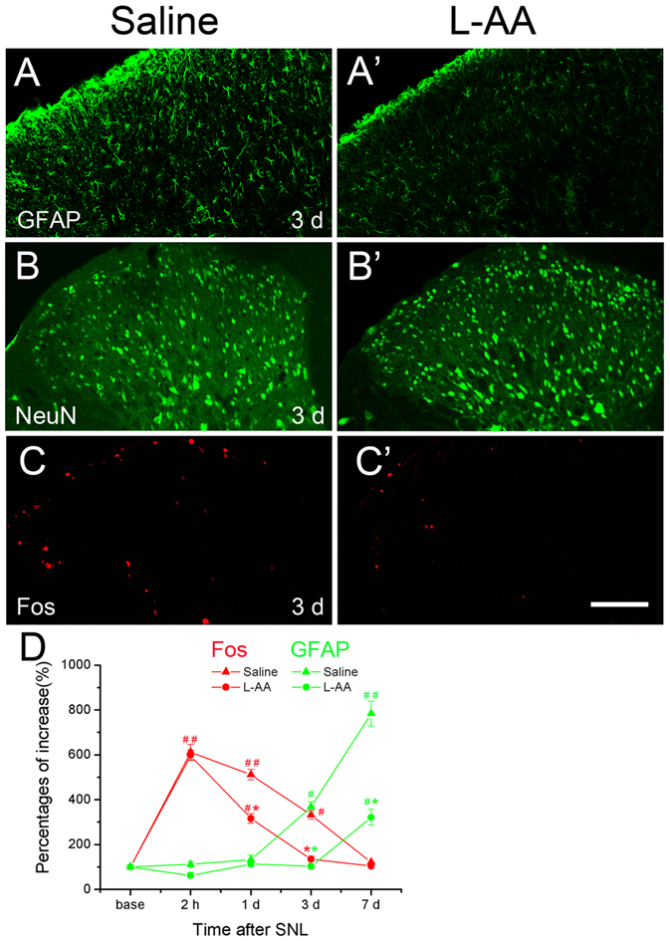
The effects of L-AA on astrocytic activation and Fos expression in the ipsilateral dorsal horn induced by SNL. Compared to the saline control group, L-AA produced a marked reduction of GFAP-positive astrocytes in the dorsal horn (A, A'). L-AA did not influence the numbers of NeuN-expressing neurons indicating the specific role of this astroglial toxin (B, B'). How ever, it did significantly suppress the Fos expression on POD 3 (C, C'). D, The changing course of astrocytic activation and Fos expression after L-AA administration to the SNL rats. The baseline level of Fos and GFAP was set as 100%, respectively. The changing course of Fos (red curves) or GFAP (green curves) was determined with comparison to the relative baseline value. L-AA postponed astrocytic activation from day 3 (saline control) to day 7. Meanwhile, this astroglial toxin shortened the duration of Fos expression from 7 d (saline control) to 3 d post SNL, although it did not change the peak level of Fos expression. #, *P*<0.05 and # #, *P*<0.01, as compared to the baseline value; *, *P*<0.05 and **, *P*<0.01, as compared to the saline control in the same time point. Scale bar = 200 µm.

### C-*fos* ASO treatment suppressed SNL-induced neuronal and astrocytic activations in different manners

Besides its effect on the SNL-induced ipsilateral mechanic allodynia, c-*fos* ASO significantly inhibited SNL-induced Fos protein expression in the ipsilateral dorsal horn. Statistical analysis revealed a significant effect of treatment as well as POD on the SNL induced dorsal horn Fos expression [Fig. 4D; two way ANOVA (between subjects factors: Group and Day): Group: F (2, 36) = 117.717, *P* = 0.000; Day: F (3, 36) = 85.247, *P* = 0.000; Group*Day: F (6, 36) = 20.944, *P* = 0.000]. Inhibition of ASO on SNL-induced Fos expression occured right after the nerve injury (at 2 h after SNL). The Fos expression level in ASO group was almost the same as baseline and this effect lasted during the whole observing window (Fig. 4D).

Meanwhile, c-*fos* ASO also inhibited the SNL-induced GFAP up-regulation but such effect was different from that on Fos expression. According to our previous study [26], SNL-enhanced GFAP expression occurs later than POD 3. Statistical analysis revealed a significant effect of treatment as well as POD on the SNL-induced GFAP up-regulation [Fig. 4D; two way ANOVA (between subjects factors: Group and Day): Group: F (2, 36) = 6.930, *P* = 0.004; Day: F (3, 36) = 103.403, *P* = 0.000; Group*Day: F(6, 36) = 2.204, *P* = 0.078]. ASO treatment rendered inhibitory effect on the enhanced GFAP on POD 3 and 7. Our changing course study of GFAP expression in the ipsilateral SDH of Saline, as well as for c-*fos* SO and ASO groups indicated that there were no obvious differences at 2 h and POD 1 when no obvious astrocytic activation could be detected (Fig. 4D). While the mean immunofluorescence density of the GFAP signal was significantly suppressed by ASO on POD 3 and 7, when the astrocytic activation in the ipsilateral dorsal horn was at a very high level in the saline control group (Fig. 4C).

### L-AA treatment down-regulated SNL-induced astrocytic and neuronal activations in different manners

Intrathecal injection of an astrocytic toxin, L-AA, produced analgesic effect on SNL-induced mechanical allodynia. As demonstrated above, this effect was significant during the late stage of the mechanic allodynia. Such behavioral change was accompanied with relevant attenuating effect on the SNL-enhanced GFAP expression. Statistical analysis revealed a significant effect of treatment as well as POD on the SNL-induced GFAP expression [Fig. 5D; two way ANOVA (between subjects factors: Group and Day): Group: F (1, 24) = 94.917, *P* = 0.000; Day: F (3, 24) = 157.417, *P* = 0.000; Group*Day: F(3, 24) = 28.865, *P* = 0.000]. L-AA treatment rendered inhibitory effect on the enhanced-GFAP on POD 3 and 7 compared to the saline control). Our changing course study of GFAP expression in the ipsilateral superficial dorsal horn of Saline and L-AA groups indicated that there were no obvious differences at 2 h and POD 1 when no obvious astrocytic activation could be detected (Fig. 5D). The effect of L-AA appeared to be astrocytic specific, because it only reduced the number of GFAP-expressing astrocytes, but not that of NeuN-expressing neurons (Fig. 5B).

To our surprise, although the total number of NeuN-positive neurons did not change under either SNL or SNL + L-AA treatment, the activation of neurons was suppressed by L-AA treatment, possibly via an indirect way other than L-AA's direct impairment to neurons. Statistical analysis revealed a significant effect of treatment as well as POD on the SNL induced SDH Fos expression [Fig. 5D; two way ANOVA (between subjects factors: Group and Day): Group: F (1, 24) = 5.284, *P* = 0.035; Day: F (3, 24) = 35.764, *P* = 0.000; Group*Day: F (3, 24) = 1.359, *P* = 0.291]. L-AA treatment rendered inhibitory effect on the enhanced Fos expression on POD 1 and 3. Studies on Fos expression in the ipsilateral superficial dorsal horn of saline controls and L-AA groups indicated that there were no obvious differences at 2 h when significant astrocytic activation could not be detected (Fig. 5D). The Fos expression of the L-AA group dropped to the normal level on POD 3, while this could only be observed on POD 7 in saline control animals. These data indicated that this astroglial toxin did not change the peak value but shorten the duration of neuronal activation (Fig. 5C, D).

In the sham-operated animals, neuronal and astrocytic activation was not influenced compared with that of naïve rats. Furthermore, intrathecal injection of c-*fos* ASO, SO or L-AA on sham-operated rats did not change the expression of Fos and GFAP (data not shown).

## Discussion

In this study, we examined the relationship between nerve injury-induced mechanical allodynia, neuronal and astrocytic activation, and there interactions. Our findings established that: (1) Inhibiting either neuronal or astrocytic activity prevented SNL induced neuropathic pain; (2) Astrocytic activation is downstream to the activation of neurons; (3) Astrocytic activation can enhance neuronal activation. Concluding from the above results, we hypothesize that there is a positive feedback circuit for neuronal-glial interaction under neuropathic pain states.

### Neuronal and astrocytic activation emerges mainly in induction and maintenance of allodynia, respectively

Allodynia is one of the clinically common symptoms which affect the patients' life quality severely. It is usually described in two different stages: induction (or initiation, usually occurs within 3 d after nerve injury in animal models) and maintenance (or duration, usually occurs later than 3 d post nerve injury).

Based on the dynamic change of Fos expression in neurons, our data suggest that neuronal activation emerges majorly in the induction of mechanical allodynia. Fos is expressed in the nuclei of nociceptive neurons following various noxious stimuli, thus serves as a marker for activated neurons. Increased Fos immunoreactivity directly reflects up-regulation in neuronal activity [19]. In our present experiment, Fos expression reached a very high level 2 h after SNL, and decreased quickly in a few days. Blocking Fos expression significantly decreased the mechanical allodynia right after the SNL. These data together, support that early increase of Fos expression is very critical for the induction of mechanical allodynia after SNL. Our point also gets literature supports in that SNL induced an immediate (<10 min) but transient (<6 h) induction of phosphorylated extracellular regulated protein kinase (pERK, marker for activated cells) restricted to neurons in the SDH, while shutdown of these activated neurons attenuated the mechanic allodynia [34].

Previous research suggested that glial cells take part in the nociceptive process with the major focus on microglia [12]. Studies on astrocytes either suggest a role downstream to microglia or less contribution to nociception [35]. While we observed that the spinal astrocytes were activated later than neurons, mainly in the maintenance of mechanic allodynia after SNL. Actually, there are still other reports that suggest a late involvement of astrocytic activation in the maintenance of nociception [17], [34].

Since neurons and astrocytes are integrated into the central nervous system (CNS), it is not correct to discuss their contribution to a certain function individually. The case for neuropathic pain is also the same. Then the next question we want to ask is whether these two major elements interact with each other for conveying nociceptive information and how?

### Neuronal activation is upstream to astrocytic activation during neuropathic pain

Since neuronal activation is earlier than that of astrocytes, it is natural to hypothesize that neuronal activation is upstream to astrocytic activation. Our data also confirmed such relationship.

In our study, c-*fos* ASO was used to knockdown the expression of Fos *in vivo*. Decreased expression of Fos was confirmed by immunofluorescent evidence and was accompanied with decreased allodynia in the early stage. In agreement with our previous study [24], c-*fos* ASO could inhibit c-*fos* activation in parallel with the decreased allodynia induced by SNL. The most remarkable inhibitory effect of c-*fos* ASO on allodynia is at the early stage after SNL, which supports the above concept: c-*fos* activation contributes more to the induction of allodynia. But a very important finding is that: Fos up-regulation can not be detected on POD 7 but c-*fos* ASO still influences allodynia at this time point. So there must be some “c-*fos*-indirectly related” factors contributing to the allodynia. Then we focused on the astrocytic activation after c-*fos* ASO treatment on SNL rats.

Very interestingly, we observed that c-*fos* ASO suppressed astrocytic activation after nerve injury. So there are at least two explanations: one is that Fos is also expressed in astrocytes and c-*fos* ASO inhibits astrocytic activation directly, and the other is that Fos is mostly expressed in neurons and the effect of c-*fos* ASO on astrocytes is indirect.

Though glial Fos expression was also observed in our experiment (Fig. 3B') and that of other investigators [36], the quantity of glial Fos expression was extremely small compared to neuronal population. We conclude that the inhibitory effect of c-*fos* ASO appeared to focus directly on neurons, suppressing neuronal activation, and further inhibiting the down stream astrocytic response indirectly. Furthermore, c*-fos* ASO only inhibited astrocytic activation but had little effect on astrocytes in their “silent” state, as shown by the invariant data for GFAP expression early after nerve injury. This observation further supports our hypothesis that the effect of c*-fos* ASO on astrocytic activation is an indirect one.

### Activated astrocytes enhance activation of their neuronal counterparts

L-AA is a cytotoxin relatively specific for astrocytes. Ultrastructural evidence suggests that cell degeneration and death is confined to astrocytes after the injection of L-AA into the striatum [37]. We have also observed that L-AA did not influence the expression of the neuronal marker NeuN. To examine whether spinal astrocytic activation is important for neuronal activation, we administrated L-AA intrathecally to SNL rats and detected Fos expression afterward. This glial toxin did not affect Fos expression early (<1 d) after nerve injury, however, it did shorten the duration of Fos expression and this change is not because of neuronal loss after L-AA application.

After chronic nerve lesions, Fos is usually expressed at an acute situation, however, its decline with time is more variable and takes much longer in different animal models. The underlying reason for this dynamic is still unexplained [20]. Our results provide here a possible reason that the duration of c-*fos* activation is likely influenced by astrocytic activity. So the different activating levels of astrocytes in pain states probably influence the dynamic of Fos duration after nerve injury. So astrocytic activation not only passively acts as the downstream of neuronal activation, but can also enhance the upstream neuronal activation, thus a positive feedback circuit probable exists between these two key elements in the spinal cord during pain states.

### A positive feedback circuit between spinal neurons and astrocytes contributes to the SNL induced neuropathic pain

Neuronal activation is followed by synthesis of various transmitters like substance P (SP), excitatory amino acids, ATP [38] and chemokines such as fractalkine [39]. Spinal astrocytic activation is “triggered” by each of these transmitters via binding to NK-1 receptors, metabotropic glutamate receptors, chemokine receptors [40], as well as purinergic receptors [41] which are expressed in astrocytes.

Receptor activation, in turn, leads to the consequent release of neuroexcitatory substances, including prostaglandins, IL-1, IL-6, and NO [42], [43] from astrocytes. As a sort of proinflammatory cytokines, interleukin-1 beta (IL-1β) is one of the most important factors which is also the focus for our literature searching.

IL-1β mediates allodynia that has the underlied potentiated neuronal nociceptive transmission [6], [18], [44]. Through binding with its receptor on the neurons, IL-1β increases calcium conductivity of neuronal NMDA receptor [45], possibly via intracellular signaling pathways leading to the phosphorylation of NMDA receptor NR1 subunit [6], [44]. Previous study [46] demonstrated that in pain state, most of the Fos is expressed in NMDA receptor-containing neurons in spinal cord. A recent study suggests that astrocyte-produced IL-1β may modulate Fos expression during pain, and the interleukin-receptor antagonist (interleukin-1ra) inhibits Fos expression and allodynia in rats [6]. Based on our literature review, a pathway of “astrocyte-IL-1β-NMDAR-Fos” may exist.

**Figure 6:**
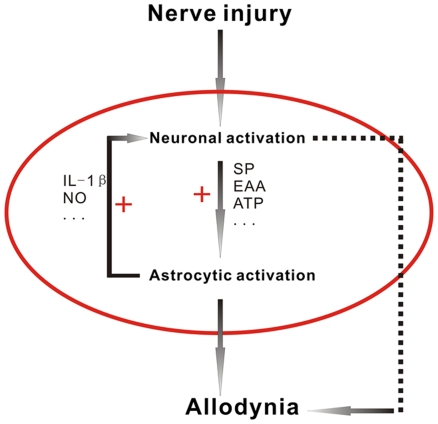
Schematic illustration of the positive feedback circuit between spinal neurons and astrocytes in neuropathic pain state. After peripheral nerve injury, various transmitters like substance P (SP), excitatory amino acids (EAA), were released from the presynaptic terminals in the superficial dorsal horn, which caused immediate (in hours) neuronal activation in the spinal dorsal horn. Neuronal activation is followed by a higher degree of synthesis and release of excitatory transmitters, which triggered late (>3 d) astrocytic activation. Activated astrocytes also produced neuroexcitatory substances, like IL-1β, IL-6, NO, and so on. These neuroexcitatory substances could action back to modify neuronal behavior, contributing to pain facilitation.

Our results indicate that injury-induced Fos expression immerges quickly, indicating a heightened activity of neurons within the dorsal horn of the spinal cord. Neuronal hyperexcitability in the pain-processing circuitry contributes to the central sensitization [2], [13]. Moreover, activated neurons synthesize and release a variety of substances that “tell” spinal astrocytes to activate [47]. And activated astrocytes also produce signaling molecules (*i.e.* IL-1β) that can action back to modify neuronal behavior, contributing to pain facilitation (Fig. 6).

While under “silent” conditions, astrocytes are primarily involved in providing energy sources and neurotransmitter precursors to neurons, cleaning up debris, regulating extracellular levels of ions and neurotransmitters [14]. Astrocytes in this state, only release very low levels of proinflammatory cytokines that is not sufficient to activate the neurons. This is probably why in our experiment, L-AA has no effect on Fos expression early after SNL.

However, we could not exclude the involvement of microglia in this kind of crosstalk. Microglia play an important role in the indirect interaction between neurons and astrocytes. We also observed that in the present study, there was apparently no astrocyte activation at 1-day post injury; yet lesions of astrocytes at 1 day attenuated neuronal activation as seen by Fos expression. It is really very interesting. We think the most probable reason is that the role of L-AA is not so specific. L-AA may influence microglial activity in some conditions. At the very early stage following nerve injury (for hours), microglia activation is obviously observed when astrocytic activation could not be detected. Microglia activity could influence neuronal activation. So besides the direct interaction, there is also indirect crosstalk between astrocytes and neurons, via microglia.

In summary, we offer evidence for the reciprocal neuronal-astrocytic signaling “dialog” underlying the mechanisms of neuropathic pain. This positive feedback circuit enlarges the effect of nerve injury on nociception and makes it more difficult to develop a clinical therapy for neuropathic pain [47]. These results further prove that non-neural elements contribute to CNS activity-dependent neuronal plasticity and behavioral hyperalgesia [13]. Said another way – they emphasize neuronal and non-neuronal elements are integrally related in nociceptive transmission, and that breaking this cycle of interaction may offer novel pain treatment strategies.

## Materials and Methods

### Animals and surgical procedures

Male adult *Sprague-Dawley* rats (180–220 g) were housed in plastic cages, three in each cage and maintained on a 12∶12 h light/dark cycle (light on at 6:00 and off at 18:00) under conditions of 22–25°C ambient temperature with food and water available. All experimental procedures received prior approval from the Animal Use and Care Committee for Research and Education of the Fourth Military Medical University (Xi'an, P. R. China) and also the ethical guidelines to investigate experimental pain in conscious animals [48]. All efforts were made to minimize animals' suffering and to reduce the number of animals used.

Under pentobarbital anesthesia (45 mg/kg, *i. p.*), the left lumbar 5 (L5) transverse process was first removed to expose the L4 and L5 spinal nerves. The L5 spinal nerve was then carefully isolated and tightly ligated with 6-0 silk thread [33]. The surgical procedure for the sham group was identical to that of the SNL group, except that spinal nerves were not ligated. For intrathecal drug administration, a midline incision (3 cm) was made at the back of the rat at the level of thoracic vertebrae 3–4. A polyethylene tube was introduced into the subarachnoid space of the lumbar enlargement. The tube was then brought out of the rat's neck skin and secured *in situ*. The rats were allowed to recover for a period of 3–5 d before further investigation. Only the animals judged as neurologically normal were used for the following experiments.

### Administration of drugs

**Figure 7:**
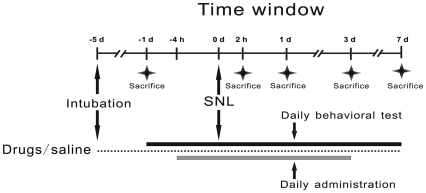
Experimental procedures. Black bar represents the period during which behavioral test was performed daily. Grey bar represents the period when drugs or saline was injected once a day. Stars represent the time points when rats were sacrificed for immunohistochemistry. Intrathecal intubation was performed on rats and followed by 4-day recovery. Behavioral test was performed 1 d before SNL to provide baseline threshold. Drugs and saline were injected 4 h prior to SNL and once a day for 3 more times after SNL. Behavioral test was performed once a day from 4 h post SNL till POD 7. SNL: spinal nerve ligation, POD: post-operation day.

The phosphorothioate-modified c-*fos* oligodeoxynucleotides were synthesized by Sangon Biotechnology Co. (Shanghai, China), and the astroglial toxin L-AA was purchased from Sigma (St. Louis, MO, USA). For c-*fos* oligodeoxynucleotides administration, the rats were randomly assigned to three groups: (1) ASO group: rats received *i. t.* injection of c-*fos* ASO (5′-GAA CAT CAT GGT CGT-3′) at a dose of 50 µg in 10 µl normal saline (n = 20); (2) SO group: rats received *i. t.* injection of c-*fos* sense oligodeoxynucleotide (SO, 5′-ACG ACC ATG ATG TTC-3′) at a dose of 50 µg in 10 µl normal saline (n = 12); (3) Saline group: rats received *i. t.* injection of rats with *i. t.* normal saline (NS, 10 µl, n = 12). For L-AA administration (n = 20), a dose of 100 nmol in 10 µl saline was *i. t.* injected, using normal saline (10 µl, n = 12) as a vehicle control. The drugs/saline were administrated daily at 4 h prior to the surgery, 1, 2, and 3 post-operation day (POD) (Fig. 7). For each injection, the solution was slowly injected within 5 min and the needle was kept for 1 more min so as to avoid the damage to the spinal cord.

### Behavioral tests

To observe how different treatments affect the allodynia, behavioral tests were performed 1 d before the drugs administration to provide baseline scores, and then from 4 h after SNL and for 1 w (once a day) to evaluate the gradual changes in the mechanical threshold of the rats. Briefly, the animals were habituated to the testing environment for 3 d before baseline testing. Animals were then put under inverted plastic boxes (30 cm×30 cm×50 cm) on an elevated mesh floor and allowed to habituate for 30 min before the threshold testing. Mechanical threshold was tested using von Frey filaments (Stoelting, Kiel, WI, USA) in a blinded manner. The ipsilateral hind paw was pressed with one of a series of von Frey filaments with gradually increasing stiffness (2, 4, 6, 8, 10, 15, and 26 g) applied to the plantar surface for 5–6 s for each filament. Each filament was applied 10 times and the minimal value which caused at least 6 responses was recorded as the paw withdrawal thresholds (PWTs). Acute withdrawal, biting, licking or shaking of the ipsilateral hind limb and vocalization were considered to be positive signs of withdrawal [49].

### Immunofluorescent staining

Animals were sacrificed at different time points (2 h, 1 d, 3 d, 7 d) after SNL. Under anesthesia with pentobarbital (60 mg/kg, *i. p.*), the rats were perfused through the ascending aorta with 100 ml of normal saline followed by 500 ml of 0.1 M phosphate buffer (PB, pH 7.3) containing 4% paraformaldehyde and 2% picric acid. After the perfusion, the L5 spinal segment was removed and postfixed in the same fixative for 2–4 h and then cryoprotected for 24 h at 4°C in 0.1 M PB containing 30% sucrose.

Transverse frozen spinal sections (30 µm thickness) were cut in a cryostat, collected serially into two dishes. Each dish contained a complete set of serial sections which were then processed for immunofluorescent staining. All of the sections in the first dish were rinsed in 0.01 M phosphate-buffered saline (PBS, pH 7.3) for three times (10 min each), blocked with 2% goat serum in 0.01 M PBS containing 0.3% Triton X-100 for 1 h at the room temperature (RT) and then used for immunofluorescent staining. The sections were incubated overnight at 4°C with primary antibodies: mouse anti-glial fibrillary acidic protein (GFAP) (1∶5000; Chemicon, Temecula, CA, USA), rabbit anti-Fos (1∶1000; Santa Cruz Biotechnology, Calif., USA) and mouse anti-NeuN (1∶3000; Chemicon), respectively. The sections were then washed for three times in 0.01 M PBS (10 min each) and then incubated for 2 h at RT with the mixture of respective secondary antibodies: FITC-conjugated horse anti-mouse IgG (1∶200; Vector, Burlingame, CA, USA) and Alexa 594-conjugated donkey anti-rabbit IgG (1∶800; Molecular Probes, Rockford, IL, USA). For double immunofluorescence of Fos/GFAP and Fos/NeuN, sections were incubated with a mixture of two primary antibodies, followed by a mixture of the two respective secondary antibodies. The specificities of the staining were tested on the sections in the second dish by omission of the specific primary antibodies. No immunoreactive products were found on the sections (data not shown). Images were obtained using a confocal laser microscope (FV1000; Olympus, Tokyo, Japan) and Digital images were captured with Fluoview 1000 (Olympus).

### Quantification and statistical analysis

Five nonadjacent sections from the L5 segments were selected randomly. In each group, 3 rats were used for statistical analysis. So in each group, there were 30 sections obtained for histology quantifications. Images were evaluated by a computer-assisted image analysis program (MetaMorph 6.1). To measure the area of GFAP immunopositive somata, we used the Threshold Image function in Measure of MetaMorph 6.1 to set the low and high thresholds for the immunofluorescent intensity which was determined to be a signal. Our image data were collected using the same region and the same size of field within same lamina to avoid any variance and difference in staining between laminae. The same configuration was used to measure cell areas in all experimental groups. The measured areas were transferred to Excel automatically for the following statistic analysis. MetaMorph 6.1 is calibrated to provide standardization of area measurements. A standardized field area was sampled arbitrarily from regions within randomly selected dorsal horn sections. And the numbers of Fos-positive cells of the same areas were counted. Then the immunoreactivities for GFAP and total numbers of Fos-immunopositive cells within the superficial dorsal horn were averaged across the five spinal sections for each experimental group [17]. Fos and NeuN double labeled neurons on the ipsilateral superficial dorsal horn were counted and the proportion of double labeled neurons to total Fos positive cells was then calculated.

All behavioral data and morphological data were colleted by experimenters blinded to the surgery and reagents treatments and statistical analyses were done by using SPSS software (version 10). Data were expressed as mean ± standard error mean (mean±S.E.M.). Two-way analysis of variance (ANOVA) (Group×Day) was used for analysis of behavioral data as well as morphological analysis. Dunnett's C *post hoc* test, assuming the variances were not equal, was employed whenever appropriate and significance was set at 0.05 for all statistical tests.
